# Identifying social perceptions of people ignoring COVID-19 warnings: a qualitative study in Iran

**DOI:** 10.1186/s13104-021-05797-0

**Published:** 2021-09-27

**Authors:** Zohreh Halvaiepour, Mehdi Nosratabadi

**Affiliations:** 1 Behavioral Sciences Research Center, Isfahan University of Medical Sciences, Isfahan, Iran; 2 Social Determinants of Health Research Center, Isfahan University of Medical Sciences, Isfahan, Iran

**Keywords:** COVID-19, Social perceptions, Qualitative study, Iran

## Abstract

**Objective:**

The COVID-19 pandemic has had various effects on the social life and daily activities of people in most countries in the world, including Iran. Hygienic precautions have been recommended, such as wearing masks and maintaining social distancing, to reduce the spread of the COVID-19. However, some people in society have not considered and ignored these health issues. This study aims to identify the sociological perceptions of people who ignore the COVID-19 warning. A qualitative study was carried out from May to July 2020. The interviewees were purposefully selected from people in Isfahan who avoided paying attention to the COVID-19 warnings. The saturation point was reached in 20 semi-structured interviews. The thematic analysis approach was used to analyze the transcribed documents using MAXQDA software (version 12).

**Results:**

The results show 2 themes and 4 sub-themes related to the sociological perception of people who ignore the COVID-19 warning. The themes and sub-themes include: feelings of social anomie (disruption and social unrest, social distrust), unmet social relationship needs (intention to maintain social participation, Feeling of reduced social support). In order to tackle social perceptions contrary to health observance during the coronavirus pandemic, educational resources such as mass media, cyberspace and social programs on the necessity and importance of health observance need to be used. Policies should also be implemented in the social, cultural and legislative contexts to enhance the degree of individuals' social responsibility.

**Supplementary Information:**

The online version contains supplementary material available at 10.1186/s13104-021-05797-0.

## Introduction

The COVID-19 pandemic occurred as a simple outbreak in Wuhan, China, in December 2019. However, it was spreading rapidly to other countries and becoming a global threat. Most countries appear to have been unprepared for this pandemic, which resulted in overcrowding in hospitals and an increase in disease-related deaths. Coronavirus has been reported in nearly 225 people. By September 10, 2021, more than 224 million people worldwide were infected and more than 4.5 million worldwide were died due to Coronavirus, A statistic which is currently risen [1]. The COVID-19 has turned into a public health pandemic [2, 3].

Governments all over the world are working hard to reduce the spread of infectious diseases like coronavirus. Mandatory masking of public places, observance of social distance, closure of assembly centers and academic institutions, providing conditions for virtual communication, and so on [4, 5].

This ensures that everybody should reduce their unwanted social contacts, unless they need to travel and work at home to avoid interpersonal transmission of the virus whenever possible. Nevertheless, studies have shown that many people choose to ignore these health requirements for financial, economic, psychological and cultural reasons [6], Nevertheless, Concerns such as decreased engagement in social interactions and decreased income due to a long-term work absence are major impediments to adopting precautionary measures [7, 8].

Many public campaigns have been developed to educate people about the coronavirus infection mechanism, but we also find that many people ignore these health warnings. In Iran, where members of society are facing serious socioeconomic challenges as a result of sanctions, preventive behavior against coronavirus is also impacted by these conditions. For example, if people are unaware of social guidelines and precautions, and for reasons such as disease stigma, cultural and behavioral factors, they may be less caring.

By conducting this research, we will be able to scientifically identify the reasons for different social groups' disobedience to disease warnings and, as a result, provide accurate recommendations to policymakers and planners. Educational and cultural interventions should also be implemented in this regard. Accordingly, the present research aims to identify these people's social perceptions and, in reality, to address the question of what are the social causes and explanations for the lack of attention towards COVID-19 from the perspective of people who do not adopt precautionary health standards?

## Main text

### Methods

A qualitative study using a conventional thematic approach was designed to investigate the People’s Social Perceptions Ignoring new Coronavirus alerts. The study was performed from May to July 2020 at Isfahan, Iran.

#### Sample and recruitment

Participants were recruited by purposeful method from Public centers in urban areas in Isfahan (such as shopping malls, urban bus and subway fleets, bakeries, and other public centers) where people do not meet the care and prevention requirements of Coronavirus. Unmasked people who did not observe social distancing were recruited, Also, people who have left home without following health guidelines, such as going to the park or traveling, for unnecessary reasons, were eligible to enter the study. People who did not obey the guidelines provided by the Ministry of Health at work were also deemed likely to enter the study if they have given reasons for not paying attention to the Coronavirus warnings in the initial and preliminary question (interview guide), they will be interviewed. Using a purposive sampling method, we reached theoretical saturation by interviewing 20 participants according to the entry criteria. Participants were selected with a maximum variety of sampling regarding age, sex, education, etc.

#### Data collection

Data were collected through interviews. The interviews were semi-structured. We derived interview guide for semi structures question from 3 in initial in-depth interview as well as literature review. A copy of the interview guide can be seen in Additional file 1.

Two researchers [M.N (PhD in Health and Social welfare) & Z.H (PhD in Psychology)] were entering public settings (such as shopping malls, bus and subway fleets, bakeries, and other public centers) and first introduced the purpose of the interview. Since qualitative research settings are real areas of experience and life, interviews are conducted in agreement with the participants and during their free time. An interview guide, informed of the research objectives, was developed (semi-structured) to explore the detailed response. In addition, in order to verify the validity of the interview guide, the interview questions were first discussed between the research team with the collaboration of an external expert and revised. The main questions of the interview started with a general question such as "(How important do you think this disease is? What should be done to prevent getting it? Or do you believe in such recommendations or not at all?). Thereafter, more exploratory questions were asked to address the interviewees’ social perceptions and the discussion phase during the interview was directed by the researcher from wider topics to smaller categories.

Each interview time lasted between 30–90 min, 40 min on average. Most interviews were tape-recorded (with the informed consent of the participants) and then transcribed verbatim.

#### Data analysis

An inductive thematic analysis was used to analyze transcribed documents assisted by MAXQDA (version 12). As a coding scheme, a conventional content analysis was conducted as follows: two authors (MN, ZH) obtained data independently coded in the first stage. For data immersion, they read and re-read the documents transcribed and listened to audio-recorded interviews. In the next phase, primary themes were extracted from the data collected and checked by the team members. Themes and sub-themes were then constructed. At the last stage, the team members checked, modified, and encoded statements.

### Results

In the current study, a total of 20 individuals were involved who ignored COVID-19 alerts. The mean age was 38.9 years (SD = 7.5) for the participants and 24 percent were female. Their level of education varied from illiteracy to the level of postgraduate education. Self-employed and entrepreneurs (n = 9), retired (n = 3), housewives (n = 3) and staffs (5) were among the participants.

Two general themes and four sub-themes were derived concerning the social expectations of individuals ignorant of the coronavirus warnings. The themes were: feeling of social anomie and social health disorder (Table 1).

**Table 1:** Themes (categories), sub-themes, and codes related to people's social perceptions ignoring new Coronavirus warnings

Theme	Sub-theme	Codes
Feeling of social anomie	Disruption and social unrest	Perception of anomaly in society
		Economic-cultural crises in society
		Irregularity and lack of binding laws
	Social distrust	Pessimism towards society
		Indifference and lack of social responsibility
Unmet social relationship needs	Intention to maintain social participation	Communication with others, institutions
		Involvement in daily social activities
	Feeling of reduced social support	Family Connections
		Support important friends/others
		Emotional support

#### Feeling of social anomie

##### Disruption and social unrest

In this regard, the interviewees stressed the existence of discrepancies, inconsistencies and the lack of binding laws in society that have generated some sort of confusion and disruption in society. Under these circumstances, the interviewees did not believe in the application of the Corona precautionary guidelines. One of the interviewees said that:

*Nobody in society seems to be obeying. There is no order, nobody is good because there are no rules, I cannot trust if these problems are true*. "

##### Social distrust

Interviewees in this field considered one of the reasons for distrust as pessimism against society as a whole, and in particular in the area of Coronavirus management.

One interviewee stated the following:

"*I do not believe it until I see it with my own eyes. These are political issues and we are all being played by the government. Corona is another of these games."*

Another sub-category relating to social distrust was indifference and lack of social responsibility. The interviewees do not care about the probability that community members will be damaged by their lack of hygiene, and in no way were considered socially responsible.

One of the interviewees stated:

*"Well, let others stay away from me, I just can't control those who are not close to me. I don't care if others have this disease.*"

#### Unmet social relationship needs

##### Intention to maintain social participation

Several concepts were identified by the interviewees who did not observe the health issues in this theme. One of these sub-categories was called Intention to maintain social participation. Within this subcategory, the interviewees expressed codes such as communication with others and interruption of day-to-day social activities. One interviewee stated the following:

*"Since this Coronavirus arrived, I was really frustrated. I can't get to my everyday commutes. I can't do my administrative work anymore as normal and I've been constrained by that."*

##### Feeling of reduced social support

Another subcategory that was relevant to “unmet social relationship needs” was the impression that interviewees had decreased social support as a result of social restrictions. Interviewees indicated reduced levels of support in family relationships, support for friendships and emotional support. One of the interviewees commented:

*“Building a relationship with my family is very important to me. I cannot end this relationship under any circumstances, even for a short time. This relationship gives me a sense of support. This corona prevents this situation. , I can't limit this relationship anymore"*

### Discussion

The study showed that two general themes, including feelings of social anomie and unmet social relationship needs (each with two subclasses) can be drawn from interviews with individuals.

Two subcategories of disruption and social unrest and social distrust have been placed on the theme of social anomie. Anomie is, in fact, a condition in a society in which there is a lack of coherence or a lack of norms and values previously shared by members of the community [9]. According to Durkheim's theory, respondents felt that this corona condition is correlated with a breakdown of norms and values, leading to the feeling that the person does not belong to others and society and is not meaningfully linked with others.

In the realm of social indifference, which is one of the categories of social anomie, people who believe that other people in society are not following the health points seem not to be required to follow those health points and believe that other people have the same views. It leads to disrespect, which creates a sense of distrust in society. Indifference is a phenomenon that affects the psychological balance of emotions, cognition, and behavior. In fact, people who are indifferent to their community health deny their commitment, civic commitment and social responsibility and do not value the health care of people in the community [10]. Numerous studies have shown a particular role in care measures for various infectious and non-communicable diseases [11, 12].

Another theme from the interviews was unmet social relationship needs, which included two sub-themes of intention to maintain social participation and Feeling of reduced social support. Participation in daily activities and contact with institutions is seen as part of the social health cycle of the person [13]. Although the main purpose of distance control and social travel restrictions is to prevent the spread of the virus at the social level and protect people at risk, these restrictions have far-reaching socio-economic impacts [14].

Participants cited the lack of social resources such as family relationships and peer support as reasons why they did not want to take preventive steps. Because family members and friends provide emotional and social support and sympathy, it can reduce anxiety and worry [15]. Social interaction decreases negative emotions like anxiety. Today, with the growing incidence of infection with COVID-19 in Iran, precautionary measures such as masking and observing social distance have led people to feel less emotional support and belonging to a group than before [16]. This contributed to the negligence of precautions.

### Conclusion

Development of educational programs about the importance of health standards on various platforms such as television, advertising, and cyberspace, as well as education and necessary warnings about the consequences and complications of the COVID-19, are among effective strategies that can be used to inform people who ignore precautionary measures. In addition, it may be useful to develop various entertainment programs in the digital space to improve family leisure time, alternative financial programs such as loans to guarantee the level of income earned before the corona, in particular those requiring daily livelihoods.

## Limitations

We have found some limitations in this study. One of the weaknesses of this study was the unique circumstances created by Corona at the community level, and it was particularly difficult for researchers to schedule and interact properly with the interviewees. Lack of face-to-face interview conditions and worries about the researcher being contaminated by interviewees due to non-compliance with health standards are among other constraints that we have encountered.

## Supplementary Information

**Additional file 1.** Interview guide.

## Data Availability

The datasets used and/or analyzed during the current study are available from the corresponding author on reasonable request.

## Abbreviation

COVID-19: Coronavirus Disease of 2019
