# Supplementary material for: Identifying social perceptions of people ignoring COVID-19 warnings: a qualitative study in Iran
Source: BMC Res Notes. 2021 Sep 27;14:382. doi: 10.1186/s13104-021-05797-0 (PMC8474853; doi:10.1186/s13104-021-05797-0)
Supplement: Supplementary file 1 — Additional file 1. Interview guide. [file 13104_2021_5797_MOESM1_ESM.docx]

**Interview guide**

Dear participant

Greetings and Regards

Thank you for taking part in this interview and for giving your time. This study is a qualitative study aimed at understanding social perceptions of ignoring the healthy warnings of COVID-19. If possible, please read the consent form to participate in the interview carefully before signing the interview, and sign it if you wish.

"If it is okay with you, I will be tape recording our conversation. I assure you that all your comments will remain confidential. If you agree to this interview and the tape recording, please sign this consent form."

I'd like to start by having you briefly describe your ideas, attitudes and issues related to COVID-19.

I'm now going to ask you some detailed questions that I would like you to answer to the best of your ability. If you do not know the answer, please say so."

**How important do you think this disease is?**

**Do you basically believe in the existence of the disease and how to spread or prevent it?**

**How has Corona affected your communication and social interactions? To what extent? Give more detailed explanation.**

**What should be done to avoid getting COVID-19?**

🡪 Instructions for the interviewer: if the person answers in a short way, the interviewer can ask about reasons for this opinion, further details about the previously mentioned measures or concepts.

**What are the main reasons why you did not heed the Corona warning?**

**What types of concerns have you had or heard regarding the social issues of COVID-19?**

**"Is there any other information about the social aspects of the COVID-19 that you think would be useful for me to know?"**

Thank you for taking part in the study. Your contribution has been invaluable and we will

be back in contact with details of any publications or papers that come out of this

research.
